# Reporting of harm in randomized controlled trials evaluating stents for percutaneous coronary intervention

**DOI:** 10.1186/1745-6215-10-29

**Published:** 2009-05-04

**Authors:** Morgane Ethgen, Isabelle Boutron, Philippe Gabriel Steg, Carine Roy, Philippe Ravaud

**Affiliations:** 1 INSERM, U738, Paris, F-75018 France; Groupe Hospitalier Bichat-Claude Bernard, Département d'Epidémiologie, Biostatistique et Recherche Clinique, Paris, F-75018 France; Université Paris VII, Faculté Xavier Bichat, Paris, F-75018 France; 2 INSERM U-698, Université Paris VII, APHP, Groupe Hospitalier Bichat-Claude Bernard, Paris, F-75018 France

## Abstract

**Background:**

The aim of this study was to assess the reporting of harm in randomized controlled trials evaluating stents for percutaneous coronary intervention.

**Methods:**

The study design was a methodological systematic review of randomized controlled trials. The data sources were MEDLINE and the Cochrane Central Register of Controlled Trials. All reports of randomized controlled trials assessing stent treatment for coronary disease published between January 1, 2003, and September 30, 2008 were selected.

A standardized abstraction form was used to extract data.

**Results:**

132 articles were analyzed. Major cardiac adverse events (death, cardiac death, myocardial infarction or stroke) were reported as primary or secondary outcomes in 107 reports (81%). However, 19% of the articles contained no data on cardiac events. The mode of data collection of adverse events was given in 29 reports (22%) and a definition of expected adverse events was provided in 47 (36%). The length of follow-up was reported in 95 reports (72%). Assessment of adverse events by an adjudication committee was described in 46 reports (35%), and adverse events were described as being followed up for 6 months in 24% of reports (n = 32), between 7 to 12 months in 42% (n = 55) and for more than 1 year in 4% (n = 5). In 115 reports (87%), numerical data on the nature of the adverse events were reported per treatment arm. Procedural complications were described in 30 articles (23%). The causality of adverse events was reported in only 4 articles.

**Conclusion:**

Several harm-related data were not adequately accounted for in articles of randomized controlled trials assessing stents for percutaneous coronary intervention.

**Trials Registration:**

Trials manuscript: 5534201182098351 (T80802P)

## Introduction

Since the development of balloon angioplasty and the introduction of stents for percutaneous coronary intervention, stent technology has rapidly evolved [1]. The technology now includes bare metal, polymer-coated, drug-eluting stents and, more recently, biodegradable and bioactive stents [2].

In fact, the first stent was implanted in humans in 1986, and by 1999, 84.2% of percutaneous coronary interventions involved stenting [1]. By the end of 2004, drug-eluting stents were used in nearly 80% of percutaneous coronary interventions in the United States, and within 3 years, several million drug-eluting stents had been implanted worldwide [1-3].

This widespread dissemination of the technology may have been achieved at the expense of insufficient assessment of harm [4-7]. Recent reports have highlighted safety concerns with drug-eluting stents, related to late adverse clinical events caused by late stent thrombosis [8]. Given the lack of large-scale randomized clinical trials with long-term follow-up of sufficient power to analyze clinical efficacy and safety of drug-eluting stents, there is an ongoing debate regarding the long-term safety of drug-eluting stents [9-15].

This debate raises the issue of the assessment and reporting of harm in randomized controlled trials evaluating stents [16]. Previous studies have shown that data pertaining to harm are underreported in articles of randomised controlled trials [17-20]. Further, compared to reports pertaining to drugs, reports of trials assessing nonpharmological treatments more often lack descriptions of harm [21]. Therefore, we aimed to assess the reporting of harm in randomized controlled trials evaluating coronary stents.

## Methods

### Search strategy and study selection

We identified all reports of RCTs assessing stents that were published between January 1, 2003, and September 30, 2008. We searched MEDLINE using the PubMed interface and the Cochrane Central Register of Controlled Trials (issue 1, 2005) using the terms *implantable device *OR *stents *[Mesh Terms] and *cardiovascular disease *[Mesh Terms] with a limitation to clinical trials published in English.

One author assessed the retrieved articles and screened the titles and abstracts to identify relevant studies. We included articles only if the study was identified as an RCT, was published as a full-text article, and assessed stents for PCI. We excluded case series, uncontrolled studies, articles published as abstracts only, editorials, news, correspondence sections, articles not including a complete description of the methods, and trials assessing other implantable devices (e.g., pacemaker, defibrillator, or cardiac valve) or stents in other vascular diseases. Reports of RCTs assessing technical interventions or surgical procedures where the use of stents was not systematically required were also excluded. We screened articles for duplicate publication (ie, the same trial published with results from different lengths of follow-up), and selected only the original articles.

### Data extraction

From a review of the relevant literature, we generated a standardized data collection form that was iterated within the research team [14,15]. Before data extraction, as a calibration exercise, two members of the team (M.E., I.B.) independently evaluated a separate set of 20 reports. A meeting followed in which the ratings were reviewed and disagreements resolved by consensus. One reviewer (M.E.) independently completed all data extractions. A second member of the team (I.B.) reviewed a random sample of 25 articles as a quality assurance exercise. The data abstraction form is available upon request.

We collected data on trial characteristics: year of publication, funding source (public support, manufacturer support or mixed), number of centers, sample size, main outcome, experimental treatment (type of stent; specific procedure of implantation such as intravascular ultrasound-guidance, and the control treatment (stent, specific procedure of stent implantation, surgery, angioplasty, pharmacological treatments or other treatments).

Gathering data for the reporting of harm involved a systematic collection of the nature of adverse events, methods used to collect harm-related information, length of follow-up, nature and severity of adverse events, adverse events causally related to treatment, and procedural complications (ie, stent loss, dissection). Since in stent trials, the main outcome is frequently a composite of cardiac death, myocardial infarction, revascularisation and/or stroke (ie, Major Adverse Cardiac Events [MACE] or Major Adverse Cardiac and (Cerebrovascular) Events [MAC(C)E]), we checked whether the harm-related data reported pertained to only MACE or MACCE, or also to other adverse events such as bleeding.

We also checked whether an adjudication committee evaluated adverse events and whether a Data Safety Monitoring Board (DSMB) was reported as being involved in the oversight of the trial, for patient safety. Data on these committees included the description of the members, whether adjudication committees were blinded and whether specific rules for submitting adverse events to the committees were reported.

Finally, as recommended by Ioannidis et al., we measured the space allocated to adverse events in the results sections (ie, proportion of lines dedicated to reporting adverse events) to evaluate the quality of reporting harm-related data [7].

### Statistical analysis

Continuous variables are reported with descriptive statistics: mean, standard deviation (SD), median (lower quartile; upper quartile) and minimum and maximum values. Categorical variables are described with frequencies and percentages.

The analysis of the space dedicated to the reporting of harm was evaluated by use of Student's t-test, Wilcoxon test or Spearman correlation as appropriate for the following factors: funding sources (manufacturer vs. public funding), type of stent (bare metal vs. polymer-coating or drug-eluting), impact factor of journal, whether the journal endorsed the CONSORT Statements, and location of the trials (North America or other countries).

All data analyses involved use of SAS for Windows, Release 9.1 (SAS Institute, Cary, North Carolina USA).

## Results

### Selected articles

We screened the titles and abstracts of 867 potentially eligible reports, examined the full text of 255 articles, and identified 132 studies meeting our inclusion criteria. The full screening process was previously published [22].

### Trial characteristics

The trial characteristics are described in Table 1. In brief, 15% of reports (n = 20) were published in a general medical journal. The median sample size was 389 patients (Q1 to Q3 109.5 – 496.5). Most trials were multicenter (48%, n = 63). The median number of centers was 15.4 (Q1 to Q3 1 – 22). However, in one-third of the reports, the number of centers involved was not reported or was unclear. The experimental treatment was described as a bare metal stent in 31% of reports (n = 41), a polymer-coated stent in 14% (n = 19), a drug-eluting stent in 49% (n = 64) and a strategy of stent use in 6% (n = 8). The control treatment was a stent in 67% of reports (n = 88) (bare metal stents in 61 reports and drug-eluting or polymer-coated stents in 27), another strategy of stent implantation in 8% (n = 10), balloon angioplasty in 18% (n = 24) and coronary artery surgery in 8% (n = 10). The source of funding was totally or partially private in almost half of the reports (42%, n = 56) but was not reported in 43% (n = 57).

**Table 1:** Reports characteristics

	n (%) n = 132
Journal	
General medical journal	20 (15.2)
Circulation	15 (11.4)
American Heart Journal	14 (10.6)
Catheter and Cardiovascular Intervention	18 (13.6)
Journal of the American College of Cardiology	17 (12.9)
American Journal of Cardiology	15 (11.4)
Other	33 (25.0)
Funding	
Public funding	16 (12.1)
Manufacturer funding	49(37.1)
Both public and manufacturer funding	7 (5.3)
No funding	3 (2.3)
Not reported	57 (43.2)
Interventions	
BMS	41 (31.1)
Polymer-coated stent	19 (14.4)
DES	64 (48.5)
Strategy of stent implantation	8 (6.1)
Comparisons (experimental intervention vs control arm)	
DES vs BMS	35 (26.5)
DES vs another DES	19 (14.4)
DES vs same DES but with a different dosage	5 (3.8)
DES vs balloon angioplasty	6 (4.5)
DES vs polymer-coated stent	3 (2.3)
DES vs surgery	1 (0.8)
	
Polymer-coated stent vs BMS	13 (9.8)
Polymer-coated stent vs angioplasty	3 (2.3)
	
BMS vs another BMS	13 (9.8)
BMS vs angioplasty	10 (7.6)
BMS vs surgery	9 (6.8)
BMS vs a strategy of stent implantation	6 (6.8)
	
Strategy of stent implantation vs another strategy of stent implantation	4 (3.0)
Strategy of stent implantation vs angioplasty	5 (3.8)

BMS = bare-metal stent

DES = drug-eluting stent

### Major cardiac adverse events

Major cardiac adverse events (death, cardiac death, myocardial infarction or stroke) were reported as the primary outcome in only 26% of reports (n = 34), as secondary outcomes (ie, clearly reported in the methods or in the results section) in 45% (n = 60) and as both primary and secondary outcomes in 10% (n = 13). In 19% of reports (n = 25), no data were provided on the rate of major cardiac adverse events.

### Assessment of harm

The reporting of the methods used to assess harm-related data is presented in Table 2.

**Table 2:** Assessment of harm described in reports of randomized controlled trials assessing stent treatment for coronary diseases by type of stent

	All reports	Drug-eluting stent	Polymer-coating stent	Bare metal stent
	N = 132	N = 64	N = 19	N = 41
	N (%)	N (%)	N (%)	N (%)
**Assessment of harm**				
- Nature of adverse events systematically collected when considering				
- All adverse events	110 (83)	50 (78)	16 (84)	39 (95)
- Events other than MAC(C)E^1^	63 (48)	22 (34)	8 (42)	16 (39)
- Mode of data collection provided when considering				
- All adverse events	29 (22)	17 (27)	5 (26)	6 (15)
- Events other than MAC(C)E^1^	13 (10)			
**Definition of expected adverse event**	47 (36)	27 (42)	2 (11)	14 (34)
				
**Assessment by an adjudication committee**	46 (35)	35 (55)	4 (21)	7 (17)
				
**Length of follow-up specified**	95 (72)	52 (81)	11 (58)	32 (78)
- < 1 month	3 (2)	1 (2)	0	2 (5)
- 6 months	32 (24)	14 (22)	7 (37)	11 (27)
- 7–11 months	27 (20)	21 (33)	1 (5)	5 (12)
- 12 months	28 (21)	14 (22)	1 (5)	13 (32)
- > 12 months	5 (4)	2 (3)	2 (11)	1 (2)
No duration specified	29 (22)	12 (19)	8 (42)	9 (22)

^1^MAC(C)E: Major Adverse Cardiac (Cerebrovascular) Event

The collection of the nature of adverse events was described in 83% of reports (n = 110), and the methods used for collecting data for at least some adverse events were given in 22% (n = 29). A definition of expected adverse events was provided in 36% of reports (n = 47), with a grading system for severity given in 7. Adverse events were adjudicated by an event committee in 35% of the reports (n = 46), with the committee membership described in 50% (n = 23/46) and the assessment of adverse events by the committee reported as blinded in 78% (n = 36/46). The specific rules for submitting adverse events to the adjudication committee were given in only six articles out of 132.

When focusing on adverse events other than cardiac or cerebrovascular events, (eg. bleeding), the systematic collection of the nature of adverse events and the method of data collection were provided in only 36% (n = 47) and 12% (n = 16) of reports, respectively.

### Length of follow-up

Adverse events were described as being followed up for less than 1 month in 2% of reports (n = 3), up to 6 months in 34% (n = 24), between 7 and 12 months in 42% (n = 55) and more than 1 year in 4% (n = 5). However, the time frame of surveillance for adverse events was not reported in 22% of reports (n = 29).

### Reporting of harm

The reporting of harm-related data is presented in Table 3. In 87% of reports (n = 87), numerical data on the nature of adverse events per treatment arm were given. These details were reported for adverse events other than MAC(C)E in half of the reports. The severity of adverse events per arm was reported with numerical data in 86% of reports (n = 114) but pertained to events other than MAC(C)E in only 13% (n = 17). Procedural complications were reported in 23% of articles (n = 30). The causality of adverse events was given in only 4 articles; adverse events were attributed to medical gestures during the implantation procedure.

**Table 3:** Reporting of harm in reports of randomized controlled trials assessing stent treatment for coronary diseases by type of stent

	All reports	Drug-eluting stent	Polymer-coating stent	Bare metal stent
	N = 132	N = 64	N = 19	N = 41
	N (%)	N (%)	N (%)	N (%)
**Nature of harm**				
- Reporting with numerical data per trial arm when considering				
- All adverse events	115 (87)	52 (81)	15 (79)	39 (95)
- Events other than MAC(C)E^1^	66 (50)	32 (50)	8 (42)	20 (49)
**Harm severity**				
- Reporting with numerical data per trial arm when considering				
- All adverse events	114 (86)	52 (81)	15 (79)	39 (95)
- Events other than MAC(C)E^1^	17 (13)			
**Relation of harm to treatment**	4 (3)	2 (3)	1 (5)	1 (2)
**Procedural complications**	30 (23)	9 (14)	3 (16)	14 (34)

^1 ^MAC(C)E: Major Adverse Cardiac (Cerebrovascular) Event

### Data Safety Monitoring Board (DSMB)

A DSMB was described in 14% of the reports (n = 18), with description of its full membership given in 15 of these (83%). The DSMB was described as adjudicating adverse events in 61% of reports (n = 11/18). In only 8 reports was the DSMB described as evaluating the totality of adverse events (serious or not). A DSMB was more often reported in trials assessing drug-eluting stents. The study sample size was higher in trials reporting than not reporting a DSMB (mean [SD] 798.3 [462.9] vs. 323.9 [357.9]).

### Space allocated to harm reporting within the articles

The mean proportion of space allocated to describing harm in the results section, the tables and figures, was 24%, 21% and 17%, respectively. The space allotted to descriptions of harm did not differ according to funding source, type of stent, journal impact factor or length of follow-up.

## Discussion

This study assessed the reporting of harm in published randomized controlled trials evaluating coronary stents published over a 6-year period. Our results suggest that harm-related data receive substantial coverage in these reports in which major cardiac adverse events are frequently evaluated as primary or secondary outcomes. However, the reporting of important issues is lacking and could be improved upon [23].

Although cardiac events are essential outcomes for trials assessing coronary stents, surprisingly, in 19% of the articles, data on these events were not reported (although they may have been collected). Further, the assessment and reporting of harm mainly focused on cardiac adverse events and neglected other events such as bleeding.

The methods used to collect data on harm and the definition of expected adverse events were insufficiently described. Only one third of reports described the evaluation of harm by an adjudication committee, but descriptions of how the committee functioned were lacking. Nevertheless, perceived differences in the treatment safety profile may result simply from the use of different methods or different definitions [17]. Determining whether a patient has reached a clinical event such as cardiac death involves some subjectivity [24]. For this reason, the US Food and Drug Administration and the European Medicine Agency recommend assessing clinical events by adjudication committees [25,26]. The importance of such committees has been outlined in several studies showing the classification of events changed in about 20% to 30% of cases after assessment by such a committee [22,27-30]. In addition, we found that the length of follow-up was short (less than 1 year in most reports), even though many case reports described late thrombosis occurring more than 14 or 20 months after implantation [16-18].

Finally, a DSMB was described in only 14% of reports. Nevertheless, a DSMB is required for clinical trials large enough to detect important effects in mortality and irreversible morbidity rates, when the risk of a treatment is unknown, and when a therapy has a known risk of severe side effects [31]. These criteria were fulfilled in most of the trial reports we selected. When a DSMB is planned, certain information concerning its governance and a monitoring plan should be provided: the DSMB should be independent of the sponsor, and its members should not have any potentially disqualifying conflicts of interest in the outcome of the trial [32]. Members with appropriate qualifications for defined roles should be chosen. However, the functioning of these committees was insufficiently described in our reports to allow for adequate appraisal.

This study has some limitations. We assessed only reports of randomized controlled trials, not the trials themselves, and failure to report is not necessarily equivalent to failure to actually carry out the procedure. Further, data on the time frame of surveying adverse events could have been reported in follow-up studies, but we focused on only the initial publication of the trial results.

## Conclusion

In conclusion, this study highlights that several harm-related data are not adequately accounted for in initial published reports of randomized controlled trials, here trials of stent treatment for coronary diseases.

## Abbreviations

BMS: bare-metal stent; DES: drug-eluting stent; DSMB: data safety monitoring board; MACE: major adverse cardiac events; MAC(C)E: major adverse cardiac and cerebrovascular events; PCI: percutaneous coronary intervention; RCT: randomized controlled trial; SD: standard deviation.

## Competing interests

The authors declare that they have no competing interests.

## Authors' contributions

Conception and design: ME, IB, CR, PhGS, PhR

Acquisition of data: ME, IB

Analysis and interpretation of data: ME, IB, CR, PGS, PR

Drafting the manuscript: ME, IB
